# Treatment coverage rates for refractive error in the National Eye Health survey

**DOI:** 10.1371/journal.pone.0175353

**Published:** 2017-04-13

**Authors:** Joshua Foreman, Jing Xie, Stuart Keel, Hugh R. Taylor, Mohamed Dirani

**Affiliations:** 1 Centre for Eye Research Australia, Royal Victorian Eye & Ear Hospital, Melbourne, Australia; 2 Ophthalmology, University of Melbourne, Department of Surgery, Melbourne, Australia; 3 Indigenous Eye Health Unit, Melbourne School of Population and Global Health, The University of Melbourne, Melbourne, Australia; Soochow University Medical College, CHINA

## Abstract

**Objective:**

To present treatment coverage rates and risk factors associated with uncorrected refractive error in Australia.

**Methods:**

Thirty population clusters were randomly selected from all geographic remoteness strata in Australia to provide samples of 1738 Indigenous Australians aged 40 years and older and 3098 non-Indigenous Australians aged 50 years and older. Presenting visual acuity was measured and those with vision loss (worse than 6/12) underwent pinhole testing and hand-held auto-refraction. Participants whose corrected visual acuity improved to be 6/12 or better were assigned as having uncorrected refractive error as the main cause of vision loss. The treatment coverage rates of refractive error were calculated (proportion of participants with refractive error that had distance correction and presenting visual acuity better than 6/12), and risk factor analysis for refractive correction was performed.

**Results:**

The refractive error treatment coverage rate in Indigenous Australians of 82.2% (95% CI 78.6–85.3) was significantly lower than in non-Indigenous Australians (93.5%, 92.0–94.8) (Odds ratio [OR] 0.51, 0.35–0.75). In Indigenous participants, remoteness (OR 0.41, 0.19–0.89 and OR 0.55, 0.35–0.85 in Outer Regional and Very Remote areas, respectively), having never undergone an eye examination (OR 0.08, 0.02–0.43) and having consulted a health worker other than an optometrist or ophthalmologist (OR 0.30, 0.11–0.84) were risk factors for low coverage. On the other hand, speaking English was a protective factor (OR 2.72, 1.13–6.45) for treatment of refractive error. Compared to non-Indigenous Australians who had an eye examination within one year, participants who had not undergone an eye examination within the past five years (OR 0.08, 0.03–0.21) or had never been examined (OR 0.05, 0.10–0.23) had lower coverage.

**Conclusion:**

Interventions that increase integrated optometry services in regional and remote Indigenous communities may improve the treatment coverage rate of refractive error. Increasing refractive error treatment coverage rates in both Indigenous and non-Indigenous Australians through at least five-yearly eye examinations and the provision of affordable spectacles will significantly reduce the national burden of vision loss in Australia.

## Introduction

Refractive error is the most readily treatable cause of vision loss, with a significant proportion of cases being correctable with spectacles or contact lenses [1]. Despite this, uncorrected refractive error is the leading cause of vision impairment (53%) and the second leading cause of blindness (21%) globally, accounting for almost 110 million cases of vision loss [2]. Given its considerable burden in both developing and developed nations, and its feasibility of treatment, uncorrected refractive error has been highly prioritised as a target for interventions in the World Health Organisation’s Vision 2020 initiative [3].

Previous population surveys conducted in the early 1990s have reported that the prevalence of uncorrected refractive error may be as high as 10% in Australians aged 40 years and older [4, 5]. While some cases of refractive error are pathological and cannot be treated with spectacles [6], the majority of cases are easily correctable, and the high prevalence of uncorrected refractive error in these studies highlights the need for increased use of spectacles by older Australians. In these studies, higher rates of uncorrected refractive error were associated with a number of demographic and environmental risk factors including country of birth, increasing age, occupation, lower socioeconomic status, and duration since last eye examination [4, 5, 7]. These barriers are considered to be surmountable through targeted interventions aimed at increasing the availability of affordable optometry services, as well as improving community awareness of these services [5, 8].

The National Indigenous Eye Health Survey (NIEHS) reported that 54% of vision loss in Indigenous Australians aged 40 years and older was caused by uncorrected refractive error, and that treatment coverage rates were consistently low in all surveyed communities [9]. However, higher spectacle coverage rates were strongly correlated with better availability of Aboriginal Medical Service (AMS) based optometry practices in communities, highlighting the importance of accessible eye healthcare services in reducing avoidable refractive vision loss [9]. Barriers to obtaining corrective spectacles in Indigenous communities are pervasive, and include insufficient service availability in remote areas and prohibitive travel distances to health services, affordability, and systematic problems with referral pathways [10].

Considering the need for improved treatment coverage of refractive error in both Indigenous and non-Indigenous Australians, a multitude of national and State-based programs have been implemented. The Australian Government’s National Framework Implementation Plan (NFIP) involves a coordinated approach to ensure equitable access to eye health care services, with improvements in refractive error treatment being highly prioritised [11]. A number of national and state-based initiatives such as the Visiting Optometrists Scheme have aimed to provide subsidised or free spectacles to improve treatment coverage rates, particularly in Indigenous Australians [10]. However, the efficacy of these programs in improving the treatment of refractive error is not currently known due to a paucity of recent population-based research, and the national treatment coverage rate of refractive error is therefore not presently known.

The National Eye health Survey (NEHS) aimed to provide up-to-date national estimates of the treatment coverage rates of refractive error in both Indigenous and non-Indigenous Australians. Here we present the treatment coverage rates for both groups across all levels of geographic remoteness and provide socio-demographic factors associated with treatment coverage.

## Materials and methods

### Ethics approval

Ethics Committee approval was obtained from the Royal Victorian Eye and Ear Hospital Human Research Ethics Committee (HREC-14/1199H). Approvals were obtained from the following state-level Indigenous ethics bodies to conduct research within Indigenous communities: Aboriginal Health and Medical Research Council of NSW (HREC-1079/15), the Menzies School of Health Research (HREC-2015-2360), the Aboriginal Health Council of Western Australia (HREC-622) and the Aboriginal Health Council of South Australia (HREC-04-15-604). Participants provided written informed consent. This study was conducted in accordance with the tenets of the Declaration of Helsinki.

### Sampling

The sampling methodology has been described in detail elsewhere [12]. In brief, data collection was conducted between March 2015 and April 2016. Probability proportional to size (PPS) multistage random cluster sampling was used to select a representative sample of Indigenous Australians aged 40 years and older and non-Indigenous Australians aged 50 years and older. Population data collected in the 2011 Australian Census were used to select 30 geographic areas across all geographic remoteness strata in Australia. Twelve Major City areas, six Inner Regional areas, six Outer Regional areas, 4 Remote areas and 2 Very Remote areas were selected. In total, 4,520 eligible non-Indigenous residents and 2240 eligible Indigenous residents were enumerated across all survey sites by trained recruiters. Of these, 3098 non-Indigenous participants (68.5% response rate) and 1738 Indigenous participants (77.6% response rate) were recruited to participate in the survey.

### Interviewer-administered general questionnaire

Recruited residents in each survey site attended a testing centre within 6km of the selected population cluster and provided written informed consent. Each participant underwent a standardised interviewer-administered questionnaire to collect key sociodemographic data, including gender, age, level of education and ethnicity. Participants were asked whether they were of Aboriginal or Torres Strait Islander origin (Indigenous Australians). The ethnicities of non-Indigenous Australians were categorised according to the Australian Standard Classification of Cultural and Ethnic Groups 2011 based on self-reported country of birth [13]. Stroke and diabetes histories were also recorded. Past ocular history was recorded for all participants, including history of diagnosis of refractive error, cataract, glaucoma, diabetic retinopathy, age-related macular degeneration, or other diseases. Participants were asked if they had ever undergone an eye examination, and if so, how recently. Those who had undergone an eye test were asked who had performed their most recent examination, and responses were recorded against a standardised list defined *a priori*: 1) Optometrist, 2) Ophthalmologist, 3) GP/local doctor, 4) Nurse, 5) Health worker, 6) Ophthalmic nurse/technician, 7) Other. Participants were asked whether they wore spectacles or contact lenses, and if so, whether their refractive correction was for distance or near correction or both.

### Eye examination protocol

A series of standardised eye examinations was performed by a trained examiner. Presenting distance visual acuity was measured in each eye separately using a logMAR chart (Brien Holden Vision Institute, Australia) at three metres in well-lit room conditions. If presenting visual acuity was worse than 6/12 in one or both eyes, pinhole testing was conducted. If vision improved to 6/12 or better in one or both eyes, auto-refraction was performed using a Nidek Ark-30 Type-R Hand-held auto-refractor/keratometer (Nidek Co. LTD, Japan). Trial lenses corresponding to auto-refraction measurements were placed in a trial frame and auto-refraction-corrected visual acuity was measured in each eye separately. Binocular presenting near vision was measured in well-lit room conditions using a CERA Vision Test E Chart (Centre for Eye Research Australia, Australia), held at the participant’s preferred reading distance. Anterior segment assessment, perimetry, intraocular pressure testing and two-field fundus photography were performed. Examiners provided participants with verbal feedback on their test results, and if abnormalities were detected, a referral letter was provided to be taken to a local doctor or optometrist.

### Definitions of uncorrected or under-corrected refractive error and refractive error treatment coverage rates

This analysis focused on participants in whom uncorrected or under-corrected refractive error was the main cause of vision loss. Uncorrected or under-corrected refractive error was determined to be the main cause of vision loss if the distance visual acuity in one or both eyes improved to better than or equal to 6/12 (≥6/12) with pinhole testing or auto-refraction. The threshold of 6/12 was selected as this is considered the legal threshold for vision impairment in Australia [14]. Participants for whom visual acuity improved with pinhole or autorefraction, but their improvement was below the 6/12 threshold remained bilaterally vision impaired, and were not included as having uncorrected or under-corrected refractive error for the purpose of this analysis. Their vision loss was primarily caused by a different condition, and adequate visual function would not be restored with refractive correction. As participants with presenting visual acuity ≥6/12 did not undergo pinhole testing or autorefraction, those individuals with ≥6/12 vision and mild refractive error who may have improved further with refraction were not identified. These participants were also not considered to have uncorrected or under-corrected refractive error.

Refractive error treatment coverage rates were calculated for participants whose vision loss was caused by uncorrected refractive error using the following formula:

Refractive error treatment coverage rate = $(\frac{n_{1}}{n_{1}+n_{2}})\times 100$. In this formula, *n*_1_ was the number of participants who reported that they wore spectacles and/or contact lenses for distance vision and achieved bilateral presenting distance visual acuity ≥6/12, and *n*_2_ was the number of participants who had refractive error as their main cause of bilateral vision loss (<6/12). Participants in *n*_2_ may have had no refractive correction (uncorrected refractive error) or they may have had refractive correction that was not sufficient to correct their visual acuity to better than 6/12 (under-corrected refractive error).

### Statistical analysis

Means and standard deviations (SD) were calculated for normally-distributed continuous sociodemographic variables, and medians and inter-quartile ranges were calculated for skewed data. Counts and percentages were calculated for categorical sociodemographic variables. Refractive error treatment coverage rates were calculated for Indigenous and non-Indigenous participants. Sampling weight-adjusted coverage rates were calculated using logistic regression models. Treatment coverage rates were disaggregated by age, gender, ethnicity, language spoken at home, and geographic remoteness, and were tabulated as counts and percentages. Multivariable logistic regression analysis was used to examine the association between spectacle or contact lens correction and the following variables: Ethnicity, including Indigenous/non-Indigenous status, age (years), gender, number of years of education, main language spoken at home (English/other), geographic remoteness time since last eye examination and the type of eye health care professional who conducted the examination. Excluding ‘optometrist’ and ‘ophthalmologist’, the *a priori* list of eye health care providers was collapsed into the group ‘other’ due to a small sample size in each group and compared against ‘optometrist’ and ‘ophthalmologist’. Due to differences in inclusion criteria and sampling between Indigenous and non-Indigenous participants, regression analysis was performed separately for each group. For the final fitted logistic regression model, model residuals and delta beta values were examined to determine if potential outlying observations influenced results. The degree to which statistical assumptions were violated was also examined. Associations were considered statistically significant if p<0.05. All statistical analyses were undertaken using Stata version 14.2 (StataCorp, College Station, TX).

## Results

### Study participants

A total of 4836 participants were recruited and examined from 30 sites across all levels of geographic remoteness in five States and one Territory in Australia. Of these, 3098 identified as non-Indigenous (46.38% male, aged 50 to 98 years, mean age [SD] = 66.6 [9.7] years), and 1738 identified as Indigenous Australians (41.1% male, aged 40 to 92 years, mean age [SD] = 55.0 [10.0] years) (Table 1).

**Table 1:** Demographic characteristics of participants with corrected refractive error and participants with uncorrected or under-corrected refractive error

	Non-Indigenous (n = 1990)		Indigenous (n = 670)	
	Uncorrected^†^	Corrected^‡^	Uncorrected^†^	Corrected^‡^
	(n = 124)	(n = 1776)	(n = 116)	(n = 554)
**Continuous variables**	**Mean (SD)**	**Mean (SD)**	**Mean (SD)**	**Mean (SD)**
Age (years)	67.6 (10.5)	67.3 (9.3)	58.5 (10.9)	57.6 (10.0)
Education (years)	11.9 (4.5)	12.7 (3.7)	10.0 (3.0)	11.0 (3.4)
**Categorical variable**	**n (%)**	**n (%)**	**n (%)**	**n (%)**
Gender (male)	62 (50.0)	753 (42.4)	46 (39.7)	205 (37.0)
English at home	116 (93.6)	1682 (94.7)	111 (95.7)	541 (97.7)
Ethnicity				
Oceanian	88 (71.0)	1265 (71.2)	116 (100.0)	553 (99.8)
European	26 (21.0)	377 (21.2)	0 (0)	1 (0.12)
Others	10 (8.1)	134 (7.6)		
Remoteness				
Major City	53 (42.7)	705 (39.7)	39 (33.6)	263 (47.5)
Inner Regional	20 (16.1)	388 (21.9)	17 (14.7)	122 (22.0)
Outer Regional	31 (25.0)	358 (20.2)	44 (37.9)	95 (17.2)
Remote	8 (6.5)	196 (11.0)	8 (6.9)	50 (9.0)
Very Remote	12 (9.7)	129 (7.2)	8 (6.9)	24 (4.3)

†Participants with uncorrected or under-corrected refractive error determined to be the main cause of bilateral presenting vision loss (<6/12)

‡Participants who reported to have distance spectacle or contact lens correction and had presenting bilateral visual acuity ≥ 6/12.

### Treatment coverage rates of refractive error and the prevalence of under- and uncorrected refractive error

**Fig 1:**
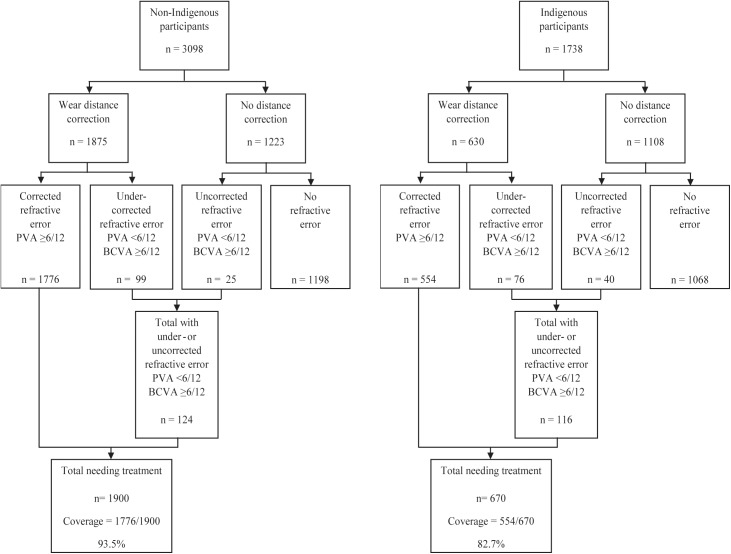
Flowcharts for treatment coverage rates for refractive error in the National Eye Health Survey. PVA = presenting visual acuity; BCVA = best-corrected visual acuity.

Distance correction was worn by 60.5% (1875/3098) of non-Indigenous participants. Uncorrected or under-corrected refractive error was determined to be the main cause of vision loss in 124 non-Indigenous Australians, resulting in a prevalence of uncorrected refractive error of 4.0%. Of these, 99 participants wore distance correction (under-corrected), while 25 did not wear distance correction (uncorrected). Overall, the number of non-Indigenous Australians with refractive correction and visual acuity ≥6/12 was 1776. The treatment coverage rate for refractive error in non-Indigenous Australians was 93.5% (1776/1900). After sampling weight-adjustment, the coverage rate remained at 93.5% (95% CI 92.0–94.8) (Table 2, Fig 1).

**Table 2:** Refractive error treatment coverage rates.

	Non-Indigenous Australians (n = 1900)				Indigenous Australians (n = 670)			
	Uncorrected^†^	Corrected^‡^	Unadjusted %	Weighted % (95% CI)^§^	Uncorrected	Corrected	Unadjusted %	Weighted % (95% CI)
Total	124	1776	93.5	93.5 (92.0–94.8)	116	554	82.7	82.2 (78.6–85.3)
Age group (years)								
40–49^¶^	**-**	**-**	-	-	29	124	81.0	79.0 (68.9–86.4)
50–59	33	390	92.2	91.0 (87.5–93.6)	35	209	85.7	86.1 (78.9–91.1)
60–69	37	701	95.0	95.3 (93.3–96.8)	34	152	81.7	79.8 (67.3–88.4)
70–79	36	476	93.0	93.5 (88.8–96.3)	14	57	80.3	83.3 (74.2–89.7)
80+	18	209	92.1	92.7 (87.0–96.1)	4	12	75.0	76.0 (35.9–94.7)
Gender								
Female	62	1023	94.3	94.0 (92.2–95.4)	70	349	83.3	82.3 (77.0–86.6)
Male	62	753	92.3	93.0 (90.4–94.9)	46	205	81.7	82.0 (75.0–87.4)
English at home								
No	8	94	92.2	93.5 (88.2–96.5)	5	13	72.2	52.7 (25.7–78.2)
Yes	116	1682	93.6	93.5 (91.9–94.9)	111	541	83.0	82.9 (78.9–86.4)
Ethnicity|^|^								
Oceanian	88	1265	93.5	93.5 (90.9–95.4)	116	553	82.7	82.2 (78.6–85.3)
European	26	377	93.6	93.6 (90.4–95.8)	0	1		-
Others	10	134	93.1	93.6 (87.2–96.9)				-
Remoteness								
Major City	53	705	93.0	93.1 (90.9–94.8)	39	263	87.1	87.2 (81.3–91.4)
Inner Regional	20	388	95.1	95.2 (93.2–96.6)	17	122	87.8	88.3 (82.8–92.2)
Outer Regional	31	358	92.0	92.1 (85.6–95.8)	44	95	68.4	69.5 (56.5–79.8)
Remote	8	196	96.1	96.7 (89.6–99.0)	8	50	86.2	87.7 (68.0–96.0)
Very Remote	12	129	91.5	91.6 (87.1–94.7)	8	24	75.0	74.6 (67.1–80.9)
Time since last eye exam								
Within 1 year	56	1167	95.4	95.6 (93.7, 96.9)	56	334	85.6	85.2 (79.4, 89.6)
1–2 years	30	415	93.3	92.1 (89.5, 94.2)	18	113	86.3	83.9 (75.5, 89.8)
2–5 years	17	159	90.3	92.7 (83.2, 97.1)	22	79	78.2	79.6 (63.1, 90.0)
≥5 years	17	31	64.6	63.2 (44.1, 78.9)	12	25	67.6	77.3 (64.0, 86.8)
Never	4	4	50.0	53.2 (18.9, 84.7)	7	3	30.0	28.4 (7.7, 65.5)

Refractive error coverage rate was defined as $(\frac{n_{1}}{n_{1}+n_{2}})\times 100$, where *n*_1_ = the number of participants who reported that they wear glasses or contact lenses for distance vision, and *n*_2_ = the number of participants with uncorrected or under-corrected refractive error as the main cause of bilateral presenting vision loss.

†number of participants with bilateral presenting vision loss (<6/12) and uncorrected or under-corrected refractive error as the main cause.

‡number of participants with refractive correction and bilateral presenting visual acuity ≥6/12.

§ Population-weighted treatment coverage rate: adjusted based on the remoteness-stratified cluster sampling protocol

¶The minimum age for non-Indigenous participants was 50 years. However, as Indigenous Australians are known to have more rapid progression and earlier onset of eye disease and diabetes, a younger age criterion of 40 years or older was selected for the target Indigenous population

||Country of birth was not disaggregated for Indigenous participants as only three individuals were born outside of Oceania.

The proportion of Indigenous participants who wore distance correction was 36.2% (630/1738). Under- or uncorrected refractive error was the main cause of vision loss in 116 Indigenous participants, of which 76 wore distance refractive correction (under-corrected) and 40 did not wear correction (uncorrected). Therefore, the prevalence of uncorrected or under-corrected refractive error was 6.7% in Indigenous Australians (116/1738), which was significantly higher than in non-Indigenous Australians (p<0.001). The total number of Indigenous participants who wore distance correction and had presenting visual acuity ≥6/12 was 554, resulting in a coverage rate for refractive error in Indigenous Australians of 82.7% (554/670). The sampling weight-adjusted coverage rate was 82.2% (95% CI 78.6–85.3) (Table 2).

### Factors associated with treatment coverage of refractive errors

Multivariable logistic regression revealed that non-Indigenous participants had a significantly higher likelihood of having adequate distance correction than Indigenous participants, with an odds ratio (OR) of 0.51 (95% CI 0.35–0.75, p = 0.001) for Indigenous participants. Due to this significant difference, as well as the different age inclusion criteria between Indigenous and non-Indigenous participants (40 years and older versus 50 years and older, respectively), all subsequent risk factor models were performed for Indigenous and non-Indigenous participants separately (Table 3).

**Table 3:** Multivariable analysis of factors associated with treatment of refractive error in Indigenous and non-Indigenous Australians.

	Non-Indigenous Australians (n = 1900)				Indigenous Australians (n = 670)			
Associated factors	Univariate		Multivariable		Univariate		Multivariable	
	OR† (95% CI)	p^*^	OR† (95% CI)	p^*^	OR† (95% CI)	p^*^	OR† (95% CI)	p^*^
Age (10 years)	1.10 (0.85–1.43)	0.45	1.17 (0.89–1.55)	0.24	0.97 (0.70–1.35)	0.85	1.04 (0.70–1.53)	0.86
Gender (male)	0.85 (0.56–1.28)	0.42	0.86 (0.54–1.37)	0.52	0.98 (0.55–1.77)	0.95	1.04 (0.57–1.89)	0.90
Education (years)	1.72 (0.76–3.93)	0.19	1.97 (0.81–4.81)	0.13	2.68 (1.33–5.42)	0.008	2.53 (0.90–7.15)	0.08
English at home	1.00 (0.49–2.05)	0.99	0.55 (0.22–1.36)	0.19	4.37 (1.22–15.67)	0.03	2.72 (1.13–6.45)	0.03
Ethnicity^‡^								
Oceanian	1		1		-	-	-	-
European	1.01 (0.55–1.85)	0.97	1.02 (0.56–1.86)	0.94	-	-	-	-
Others	1.01 (0.40–2.58)	0.98	0.78 (0.23–2.68)	0.68	-	-	-	-
Remoteness								
Major City	1		1		1		1	
Inner Regional	0.54 (0.34–0.86)	0.11	1.35 (0.75–2.44)	0.31	1.11 (0.59–2.10)	0.73	1.01 (0.51–2.02)	0.97
Outer Regional	0.59 (0.22–1.61)	0.69	0.89 (0.46–1.73)	0.72	0.33 (0.16–0.68)	0.004	0.41 (0.19–0.89)	0.03
Remote	0.08 (0.03–0.19)	0.22	2.34 (0.62–8.91)	0.20	1.05 (0.29–3.78)	0.94	1.39 (0.38–5.03)	0.60
Very Remote	0.05 (0.01–0.27)	0.47	0.90 (0.48–1.67)	0.72	0.43 (0.24–0.77)	0.006	0.55 (0.35–0.85)	0.01
Time since last eye exam								
Within 1 year	1		1		1		1	
1–2 years	0.54 (0.34–0.86)	0.01	0.49 (0.32–0.76)	0.002	0.91 (0.47–1.75)	0.76	0.93 (0.51–2.02)	0.81
2–5 years	0.59 (0.22–1.61)	0.29	0.58 (0.22–1.53)	0.26	0.68 (0.24–1.96)	0.46	0.69 (0.23–2.01)	0.49
≥ 5 years	0.08 (0.03–0.19)	<0.001	0.08 (0.03–0.21)	<0.001	0.59 (0.27–1.31)	0.19	0.70 (0.32–1.54)	0.36
Never	0.05 (0.01–0.27)	0.001	0.05 (0.01–0.23)	0.001	0.07 (0.11–0.43)	0.006	0.08 (0.02–0.43)	0.004
Examined by								
Optometrist	1		1		1		1	
Ophthalmologist	0.62 (0.31–1.24)	0.17	0.49 (0.25–0.95)	0.06	0.68 (0.41–1.13)	0.13	0.81 (0.48–1.38)	0.43
Others	0.19 (0.03–1.00)	0.05	0.19 (0.03–1.09)	0.06	0.25 (0.10–0.61)	0.004	0.30 (0.11–0.84)	0.02

†Odds ratio for the association between factors and treatment of refractive error. CI = Confidence Interval

‡ Country of birth was not tested for Indigenous participants as only three individuals were born outside of Oceania

*Statistical significance was set as a p value of <0.05 (two tailed).

In non-Indigenous participants, treatment coverage rates did not vary significantly by age or gender (Table 3). Non-Indigenous Australians who reported that they had not undergone an eye examination within the past five years were less likely to have had their refractive error corrected (OR 0.08, for ≥5 years, and 0.05, for those who had never been examined). Participants who reported that they had seen an ophthalmologist were less likely to have had their refractive error corrected (OR 0.49) compared to those who had seen an optometrist.

Numerous factors were associated with a lower likelihood of having corrected refractive error in Indigenous participants. With treatment coverage rates of 68.4% in Outer Regional (OR 0.41) and 75% in Very Remote (OR 0.55) sites compared to the highest rate of 87.2% in Major Cities, geographic remoteness was a risk factor for having under- or un-corrected refractive error. Having never undergone an eye examination (OR 0.08) and having visited a health worker other than an optometrist or ophthalmologist for the most recent eye examination (OR 0.30) were also associated with a decreased likelihood of corrected refractive error in Indigenous participants. Conversely, speaking English at home conferred an increased likelihood of having corrected refractive error (OR 2.72).

## Discussion

This paper provides the treatment coverage rates of uncorrected refractive error and associated risk factors in Australia’s first National Eye Health Survey. Treatment coverage rates were above 90% in non-Indigenous Australians of all ages, and of both genders residing in all levels of geographic remoteness. Conversely, the refractive error treatment coverage rate in Indigenous Australians varied considerably according to sociodemographic risk factors including spoken language and geographic remoteness, and was significantly lower than that of non-Indigenous Australians.

The high refractive error treatment coverage rate of 93.5% in non-Indigenous Australians aged 50 years and older may be explained by widespread accessibility of spectacle-dispensing optometry services. Previously, two surveys in the early 1990s, the Blue Mountains Eye Study (BMES) and the Melbourne Visual Impairment Project (VIP) elucidated that under- and uncorrected refractive error were pervasive, with 45.6% of participants in the BMES and 57% in the VIP having correctable presenting visual acuity [4, 5]. Comparability between the NEHS and these studies is restricted by differences in definitions of under- or uncorrected refractive error, with the BMES defining under-corrected refractive error as an improvement of two or more lines on a logMAR chart in those with visual acuity 6/9 or worse [5] and the VIP considering under-corrected refractive error as an improvement on one or more lines in those with visual acuity worse than 6/6 minus two letters [4]. As such, other parameters including the prevalence of uncorrected refractive error and temporal changes in risk factors must be compared with similar caution. Nonetheless, current national strategies for spectacle coverage appear, on the whole, to have improved coverage in non-Indigenous Australians [11]. Where those with poorer education and those of older age were previously shown to be at risk of uncorrected refractive error, the lack of these associations in the NEHS suggests that improvements in availability and utilisation of optometry services in recent years may have contributed to closing these gaps. Interestingly, despite previous reports that regional and remote Australia were severely under-serviced by optometrists, coverage rates in non-Indigenous participants in our study were stable across remoteness strata, suggesting that outreach programs that incentivise optometrists to practice in remote areas are proving effective for non-Indigenous Australians [15, 16]. Indeed, previous Australian research has suggested that non-Indigenous Australians residing in non-urban areas undergo more frequent optometric examinations than their urban counterparts [17]

The high treatment coverage rate in non-Indigenous participants should be considered in light of the fact that uncorrected refractive error is Australia’s leading cause of vision impairment, with even small rates of under-correction (or no correction) contributing significantly to the national burden of vision loss [7, 18]. Indeed, 4% of all non-Indigenous participants had uncorrected refractive error as a main cause of vision loss, corresponding to more than 200,000 Australians. The finding that those who had not undergone an eye examination within the past five years were at risk for uncorrected refractive error supports previous Australian research and highlights the need for older Australians (specifically those without previously diagnosed eye disease) to undergo a vision assessment approximately every five years [8, 19]. The resulting reduction in the burden of uncorrected refractive error may prove to be a highly cost-effective and efficient method to reduce avoidable vision loss as a public health concern in Australia [14].

The disparity in the treatment coverage rate between Indigenous and non-Indigenous Australians suggests that Indigenous Australians are comparatively under-serviced with eye health care resources. While outreach services have been initiated to close the gap in Indigenous eye health by providing free or subsidised spectacles, previously identified coordination problems and funding deficits may contribute to the lower coverage rate in Indigenous communities [10, 20, 21]. One logistical problem of particular concern that was supported by the results of our survey was the lack of accessibility to spectacles by Indigenous Australians living in remote parts of Australia, with the lowest rates in Outer Regional and Very Remote sites [9, 21]. A well-coordinated and integrated approach involving improvements in availability and utilisation of services in these under-serviced regions, as outlined in the Roadmap to Close the Gap for Vision, is required to improve treatment coverage rates [20]. As Turner et al. showed [9], increasing the availability of optometry services in Indigenous communities is insufficient to increase spectacle coverage rates. However, when increased optometry services were hosted by local AMSs, spectacle coverage rates increased significantly. Therefore, care pathways aiming to improve spectacle coverage in remote communities should increase the role of AMSs in identifying those in need of spectacles and increasing the frequency with which outreach optometry services operate within AMS practices to provide culturally-appropriate services. We also identified that Indigenous participants who received their last eye examination by a health worker other than an optometrist or an ophthalmologist were at greater risk of uncorrected refractive error. This highlights both the indispensable utility of optometrists and ophthalmologists, as well as the need for sustainable models that continue to improve the education and training of health workers in Indigenous communities.

The strengths of this study include; 1) the sampling methodology and large sample size obtained that allowed robust extrapolation of findings to the Australian population, and 2) the comprehensive questionnaire that allowed us to identify important risk factors for uncorrected refractive error. A potential limitation of the study is that we did not ascertain a history of refractive surgery as a treatment for refractive error. This may have resulted in a slight under-estimation of the true treatment coverage rate. Another limitation may be that hand-held autorefraction has been shown to be less accurate than table-mounted autorefraction [22] and subjective refraction [23], and the use of a hand-held autorefractor may have produced inaccurate results in some participants. Nonetheless, the accuracy of hand-held autorefractors has been shown to be sufficient for screening purposes, [22, 24] suggesting that these inaccuracies are unlikely to have substantially affected treatment coverage estimates.

In conclusion, the treatment coverage rate of refractive error was significantly higher in non-Indigenous Australians than in Indigenous Australians. Indigenous Australians living in more remote areas, those who have never had an eye examination, and those who utilise non-optometric eye care services, were at high risk for untreated refractive error. Improvements in AMS-mediated optometry services in remote communities and the provision of affordable spectacles in Indigenous communities are required to increase treatment rates. Given that uncorrected refractive error accounts for a significant proportion of vision loss in Australia, ensuring that older Australians undergo eye examinations every five years will contribute substantially to reducing the burden of vision loss in Australia.
